# Afterload-related cardiac performance: a hemodynamic parameter with prognostic relevance in patients with sepsis in the Emergency Department

**DOI:** 10.1186/cc9491

**Published:** 2011-03-11

**Authors:** J Wilhelm, S Hettwer, M Schürmann, S Bagger, F Gerhardt, S Mundt, S Muschick, J Zimmermann, H Ebelt, K Werdan

**Affiliations:** 1 Martin-Luther-University, Halle (Saale), Germany

## Introduction

Afterload-related cardiac performance (ACP) was developed to describe cardiac function in patients with sepsis, when cardiac output (CO) is increased due to a decline in systemic vascular resistance (SVR). We now studied the prognostic relevance of ACP in comparison with the cardiac index (CI) and cardiac power index (CPI) in patients at a very early stage of community-acquired sepsis (CAS) in the Emergency Department.

## Methods

In patients ≥18 years admitted to our Emergency Department with CAS (infection and ≥2 SIRS criteria), CI, CPI, and ACP were measured either non-invasively (TaskForce-Monitor; CNSystems, Austria) or invasively. ACP was calculated as ACP = 100 × CO/(560.68 × SVR^-0.64^). Cardiac function was graded into normal (> 80%), slightly (61 to 80%), moderately (41 to 60%) or severely impaired (≤40%).

## Results

**Figure 1:**
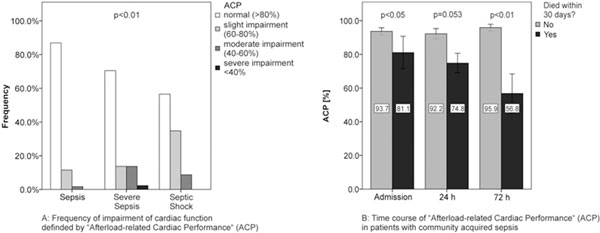
**(A) Frequency of impairment of cardiac function defined by ACP**. **(B) **Time course of ACP patients with CAS.

Of 137 patients studied, 48.2% had sepsis, 33.6% severe sepsis, and 18.2% septic shock. Overall 30-day mortality was 10.9%. On admission ACP was 86.7 ± 27.7% in severe sepsis and 85.5 ± 25.8% in septic shock, significantly lower than in patients with sepsis without signs of organ dysfunctions (98.6 ± 22.3%, *P *< 0.01), whereas no differences were observed for CI or CPI, respectively. In severe sepsis or septic shock, impairment of ACP was observed more often than in sepsis (Figure 1A). Nonsurvivors showed a significantly depressed ACP already on admission and after 72 hours (Figure 1B), whereas CPI differed only after 72 hours between survivors and nonsurvivors (0.52 ± 0.18 vs. 0.32 ± 0.17, *P *< 0.05) and CI showed no differences in this regard. ACP correlated better with APACHE II score (*r *= -0.371, *P *< 0.001) than CPI (*r *= -0.330, *P *< 0.001) or CI (*r *= -0.220, *P *< 0.001). Only ACP correlated with serum levels of procalcitonin (*r *= 0.224, *P *< 0.01) and IL-6 (*r *= -0.173, *P *< 0.05).

## Conclusions

Taken together, only the parameter ACP but not CI nor CPI is able to detect an early impairment of cardiac function in patients with CAS and provides prognostic information on admission.

